# Metastatic castrate-resistant prostate cancer with a late, complete and durable response to docetaxel chemotherapy: a case report

**DOI:** 10.1186/1752-1947-8-122

**Published:** 2014-04-09

**Authors:** Luis Daverede, Christy Ralph, Satinder P Jagdev, Ioannis Trigonis, Sebastian Trainor, Patricia Harnden, Michael Weston, Alan Paul, Naveen S Vasudev

**Affiliations:** 1 Department of Medical Oncology, St James’s Institute of Oncology, Leeds LS9 7TF, UK; 2 Department of Pathology, St James’s Institute of Oncology, Leeds LS9 7TF, UK; 3 Department of Radiology, St James’s Institute of Oncology, Leeds LS9 7TF, UK; 4 Department of Urological Oncology, Paul Sykes Centre, St James’s University Hospital, Leeds LS9 7TF, UK; 5 Cancer Research UK Centre, Leeds Institute of Cancer Studies and Pathology, Leeds LS9 7TF, UK

**Keywords:** Castrate-resistant prostate cancer, Chemotherapy, Complete response, PSA

## Abstract

**Introduction:**

Although treatment options for men with metastatic castrate-resistant prostate cancer have improved in recent years, the outlook for patients remains poor, with overall survival in the region of 2 years. Response rates with chemotherapy are modest and disease progression is usually observed within months of stopping treatment.

**Case presentation:**

We present a case of a 72-year-old White man of British origin with metastatic castrate-resistant prostate cancer with bulky lymphadenopathy and a serum prostate-specific antigen of 295μg/L. He received treatment with docetaxel chemotherapy plus prednisolone, but received just 3 cycles before treatment was stopped due to toxicity and lack of response (prostate-specific antigen was 276μg/L 4 weeks after the last dose and there was a confirmed stable appearance on computed tomography scan). Unexpectedly, at follow-up 4 months later, the patient was clinically better; his prostate-specific antigen had dramatically improved to 4.1μg/L and a re-staging computed tomography scan revealed complete resolution of his bulky lymphadenopathy. At the time, he was receiving a luteinising hormone-releasing hormone analogue but no other disease-modulating treatment. He remains well and asymptomatic, with his most recent serum prostate-specific antigen measuring 0.14μg/L, 18 months after last receiving chemotherapy.

**Conclusion:**

We report a case of complete and durable regression of metastatic castrate-resistant prostate cancer following palliative chemotherapy which, to the best of our knowledge, has not previously been reported in the literature.

## Introduction

Prostate cancer is a disease of increasing significance worldwide. In many industrialised nations, it is one of the most common cancers and among the leading causes of cancer deaths [1]. In the United Kingdom, there are >40,000 new cases diagnosed each year, accounting for 25% of new cases of cancer in males [2].

Treatment for men with metastatic prostate cancer is given with palliative intent. Androgen deprivation therapy, using either surgical castration or gonadotrophin analogues/anti-androgens, achieves a period of disease control in most men, typically lasting between 2 and 3 years. At this point, tumours are able to proliferate despite castrate levels of androgens; this is commonly referred to as castrate-resistant prostate cancer (CRPC).

Until recently, few treatment options improved survival in men with metastatic CRPC. Chemotherapy, using docetaxel (75mg/m^2^ every 3 weeks) and oral prednisolone (5mg twice daily), remains a mainstay of treatment, having been established almost a decade ago [3]. However, evidence supporting the use of other, more novel, hormonal agents such as the androgen biosynthesis inhibitor abiraterone acetate [4] and the novel androgen receptor signalling inhibitor enzalutamide [5] now exist.

Docetaxel chemotherapy, typically given as a block of six to 10 cycles, is associated with a modest 2 to 3 month median survival advantage, and overall survival, even in the setting of recent clinical trials, remains less than 2 years [6]. Prostate-specific antigen (PSA) response rates, defined as a 50% reduction, are between 45 and 64% and Response Evaluation Criteria In Solid Tumours (RECIST)-defined [7] radiological response rates are between 12 and 28% [3,6]. However, even when responses are observed, they are rarely durable, with progression typically observed at a median of 6 to 8 months [3,6].

## Case presentation

**Figure 1:**
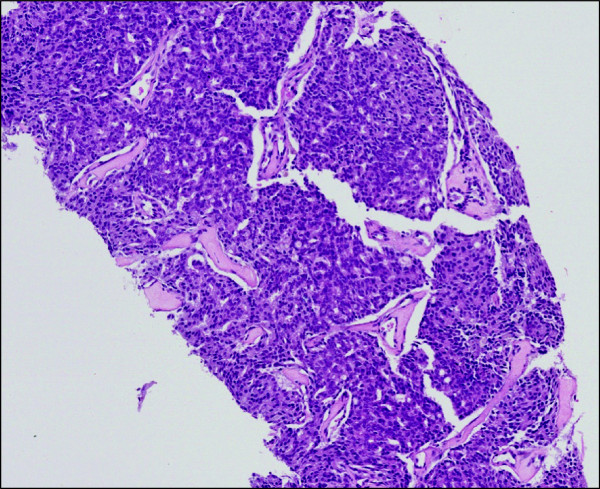
**Histology from prostate biopsy.** Core biopsies of the patient’s prostate gland at diagnosis revealed a typical prostatic adenocarcinoma, Gleason score 4+4=8. Hematoxylin and eosin-stained section.

A 72-year-old White man of British origin presented in December 2004 with an elevated serum PSA of 21μg/L (reference range <5.0μg/L) detected in primary care. A prostate biopsy revealed a Gleason score of 8 (4+4) prostatic adenocarcinoma (Figure 1), following expert pathological review. A computed tomography (CT) scan and bone scintigraphy performed in January 2005 showed no evidence of local spread or distant metastases.

Significant medical history included hypertension. His medications at diagnosis were irbesartan and felodipine.

**Figure 2:**
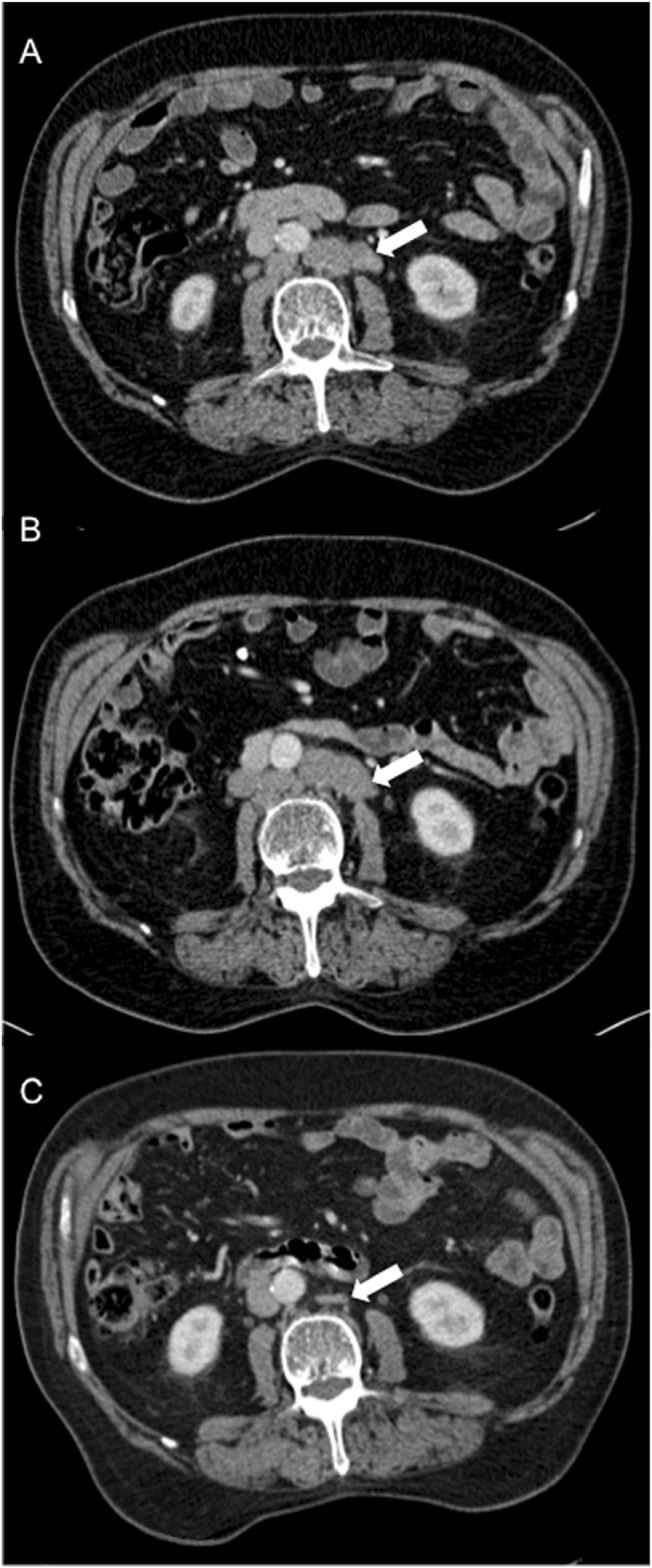
**Serial abdominal computed tomography scans. (A)** Baseline computed tomography scan performed 3 days prior to the start of chemotherapy. Bulky retroperitoneal lymph nodes are visible (arrow). **(B)** End-of-treatment computed tomography scan performed 28 days following the last dose of chemotherapy. The lymphadenopathy persists (arrow) and was deemed stable by Response Evaluation Criteria In Solid Tumours criteria. **(C)** Follow-up computed tomography scan, performed 203 days following the last dose of chemotherapy. This scan was requested in view of the dramatic fall in PSA. Complete resolution of the lymphadenopathy is observed, with only a small residuum left (arrow).

Despite high-risk disease, he declined radical treatment. Therefore magnetic resonance imaging staging was not performed and he was initially managed with active surveillance. In September 2005, due to a PSA rise to 57μg/L, he was commenced on anti-androgen monotherapy with bicalutamide, achieving a PSA nadir of 0.5μg/L 2 years later. In July 2011, almost 6 years after commencing therapy, his PSA increased to 48μg/L. Bicalutamide was withdrawn and the luteinising hormone-releasing hormone (LHRH) analogue goserelin (10.8mg intramuscular 3-monthly) was initiated. Due to a lack of PSA response, maximum androgen blockade was commenced 3 months later, with the addition of bicalutamide. His serum PSA continued to rise and reached 269μg/L in March 2012. A serum testosterone level at this time, of less than 0.3nmol/L (normal range 8.0 to 30nmol/L), confirmed medical castration. He was therefore considered for palliative chemotherapy. His only symptom at this stage was lethargy. A re-staging CT scan in April 2012 showed multiple enlarged lymph nodes in his mid and lower retroperitoneum measuring up to 3.5cm (Figure 2A), as well as in his mediastinum and left supraclavicular fossa, consistent with metastatic prostate carcinoma. Bone scintigraphy remained negative. Blood tests showed: haemoglobin level 12.4g/L (normal range 13.5 to 18.0g/L); white cell count 8.19×10^9^/L (normal range 4.00 to 11.00×10^9^/L); platelets 302×10^9^/L (normal range 150 to 400×10^9^/L); alkaline phosphatase 246IU/L (normal range 70 to 300IU/L).

The patient received three cycles of 3-weekly docetaxel at 80% of our standard dose (75mg/m^2^) and prednisolone (5mg twice daily) from April to June 2012. During this time his medications were felodipine 2.5mg once a day, eprosartan 150mg once a day, simvastatin 40mg once a day, calcium supplements and goserelin 10.8mg intramuscular 3-monthly. Chemotherapy was stopped due to grade 3 fatigue and a lack of PSA response (295μg/L immediately pre-chemotherapy and 276μg/L 4 weeks after the last dose was given). A re-staging CT scan confirmed a lack of a radiological response (Figure 2B, arrow), classified as stable disease as per RECIST 1.1 [7].

At review 4 weeks following his last cycle of chemotherapy, the patient complained of shortness of breath, palpitations and fatigue. He was referred to cardiology and an echocardiogram and 24-hour ambulatory electrocardiogram (ECG) were requested. The echocardiogram was unremarkable and the 24-hour ECG confirmed a brief episode of paroxysmal atrial fibrillation. He was therefore commenced on aspirin 75mg and bisoprolol 2.5mg daily.

**Figure 3:**
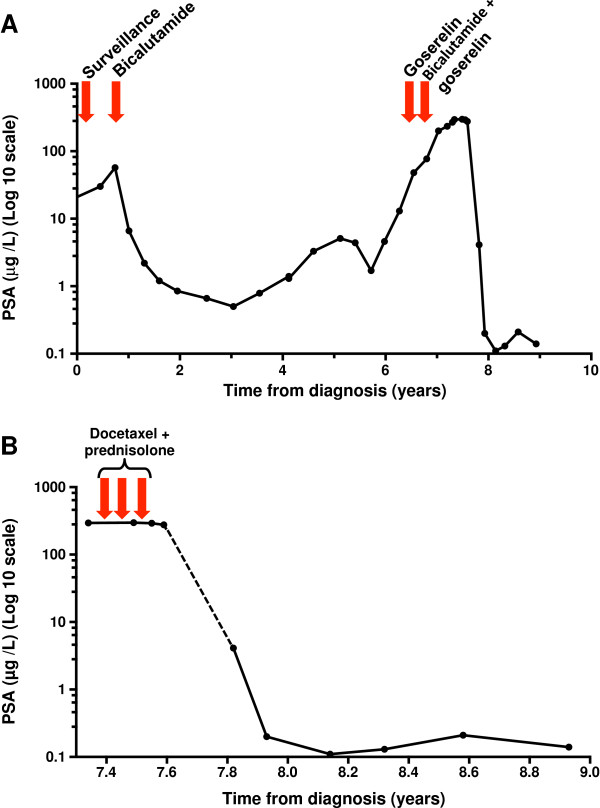
**Line plots of change in serum prostate-specific antigen with time. (A)** Change in serum prostate-specific antigen from diagnosis (log 10 scale). The patient achieved almost 6 years of disease control with androgen-deprivation therapy before developing a castrate-resistant state. The prostate-specific antigen can be seen to initially rise despite bicalutamide, then subsequently to rise despite a switch to goserelin, and finally despite maximum androgen blockade (bicalutamide plus goserelin). The patient then went on to receive three cycles of docetaxel plus prednisolone chemotherapy. A dramatic and sustained fall in prostate-specific antigen was observed 4 months after last receiving chemotherapy. This section of the plot has been expanded in Figure 3**B** for clarity. **(B)** Change in serum prostate-specific antigen (log 10 scale) in relation to chemotherapy. The prostate-specific antigen remained stable during chemotherapy (>250μg/L) and for at least 34 days following the last dose. However, at follow-up 4 months following his last dose of chemotherapy, his serum prostate-specific antigen had fallen to 4.1μg/L. When last seen, his latest prostate-specific antigen reading was 0.14μg/L, 18 months since his last cycle of chemotherapy. Abbreviation: PSA, prostate-specific antigen.

Unexpectedly, 4 months following the last cycle of chemotherapy, his performance status score and lethargy had improved dramatically and his PSA had fallen to 4.1μg/L. A re-staging CT scan in December 2012 (Figure 2C) showed a complete resolution of the substantial lymphadenopathy at all sites. Over the following months his serum PSA continued to decline and measured 0.14μg/L 18 months following his last cycle of chemotherapy. The 3-monthly injections of goserelin, introduced approximately 10 months prior to commencing chemotherapy, have never been interrupted. He remains completely asymptomatic with a sustained normalisation of his serum PSA. Alterations in serum PSA levels from diagnosis and their correlation with treatment events are illustrated in Figure 3A and 3B.

## Discussion

Complete and durable regression of metastatic CRPC is rare, and to the best of our knowledge it has not previously been reported in the literature. We believe this represents a late, complete and durable response to docetaxel chemotherapy.

Our patient had histologically proven Gleason 8 prostatic adenocarcinoma at diagnosis. At the time of starting chemotherapy, he had documented CRPC, with substantial lymphadenopathy and a PSA >250μg/L. He received just three cycles of reduced-dose docetaxel plus prednisolone with documented stable disease as best response at the end of therapy, based on PSA and cross-sectional imaging (4 weeks following the last cycle of chemotherapy). His serum PSA was not checked again until 3 months later when it was found to have almost normalised. We cannot be certain therefore at exactly what point in time after chemotherapy his PSA began to fall.

Late responses to docetaxel chemotherapy in CRPC are recognised and reductions in serum PSA are sometimes not observed until cycle four of treatment [8]. Equally, normalisation of serum PSA can occur with chemotherapy. However, the duration of this response (currently 18 months) with accompanied complete response on cross-sectional imaging, following just three cycles of chemotherapy, makes this case unusual.

At the point at which the patient’s cancer was noted to be regressing, the only treatment he was receiving for his prostate cancer was an LHRH-agonist, an agent on which he had previous documented disease progression, based on serial PSA and CT scans. Other medications he received or was receiving included felodipine, irbesartan, eprosartan, calcium tablets, bisoprolol, simvastatin and aspirin. Pre-clinical data supporting the anti-neoplastic effects of simvastatin on prostate cancer cells exist [9], but whether statins influence the risk of developing prostate cancer [10] or are beneficial for patients with metastatic disease remains uncertain. Aspirin has been associated with a reduced risk of prostate cancer-specific mortality in men treated with radical radiotherapy or radical prostatectomy [11], but its activity in patients with advanced disease is also unknown. Our patient had only received 7 days of aspirin before his serum PSA was found to have normalised, and is therefore of questionable relevance.

Complete remissions to hormonal manipulation have been described in patients with prostate cancer [12,13], but this seems an unlikely mechanism in this case. Our patient had a prolonged initial response to anti-androgen therapy (>5 years), but subsequent withdrawal of anti-androgen and replacement with an LHRH-agonist led to continued disease progression. Thus it seems unlikely that, although the same anti-androgen was later re-instigated and subsequently stopped at the time of initiation of chemotherapy, anti-androgen withdrawal is responsible for the observed response.

Finally, we acknowledge that in this patient, presenting with high-grade (Gleason 8) cancer, relapse with more de-differentiated, non-PSA secreting, disease may occur. We consider this unlikely at the present time given that the patient remains so well but this cannot be excluded since we have not recently repeated cross-sectional imaging. Our current approach would be to only do this based on clinical symptoms, in the absence of a PSA rise.

## Conclusions

We report a case of a patient with metastatic CRPC, with bulky lymph nodes and a serum PSA >250μg/L, in which the disease was observed to regress completely on cross-sectional imaging, combined with PSA normalisation, 4 months after receiving three cycles of docetaxel chemotherapy plus prednisolone. The patient remains well with normal serum PSA (0.14μg/L), 18 months after last receiving chemotherapy. We believe this to represent a delayed, complete and durable response to docetaxel chemotherapy which is extremely rare.

## Consent

Written informed consent was obtained from the patient for publication of this case report and accompanying images. A copy of the written consent is available for review by the Editor-in-Chief of this journal.

## Abbreviations

CRPC: Castrate-resistant prostate cancer; CT: Computed tomography; ECG: Electrocardiogram; LHRH: Luteinising hormone-releasing hormone; PSA: Prostate-specific antigen; RECIST: Response Evaluation Criteria In Solid Tumours.

## Competing interests

The authors declare that they have no competing interests.

## Authors’ contributions

LD, CR, SJ, IT, ST, AP and NV contributed to the clinical management of the patient during his treatment and subsequent follow-up. PH and MW provided expert pathological and radiological review respectively. LD and NV wrote the initial draft, which was critically appraised by all authors. All authors read and approved the final manuscript.
